# Correction: Preparation of magnesium silicate/carbon composite for adsorption of rhodamine B

**DOI:** 10.1039/c8ra90019a

**Published:** 2018-02-26

**Authors:** Zhiwei Sun, Xinhui Duan, C. Srinivasakannan, Jinsheng Liang

**Affiliations:** Key Laboratory of Special Functional Materials for Ecological Environment and Information, Hebei University of Technology, Ministry of Education Tianjin 300130 China liangjinsheng@hebut.edu.cn; Institute of Power Source and Ecomaterials Science, Hebei University of Technology Tianjin 300130 China; Chemical Engineering Department, Khalifa University of Science and Technology, The Petroleum Institute Abu Dhabi United Arab Emirates

## Abstract

Correction for ‘Preparation of magnesium silicate/carbon composite for adsorption of rhodamine B’ by Zhiwei Sun *et al.*, *RSC Adv.*, 2018, **8**, 7873–7882.

Table 4 in the published article was incomplete; a revised table with added Δ*G* values is reproduced below:

**Table 1:** Thermodynamic parameters of the adsorption of RhB onto magnesium silicate/carbon composite

*T* (K)	Δ*G*^0^ (kJ mol^−1^)	Δ*H*^0^ (kJ mol^−1^)	Δ*S*^0^ (J mol^−1^ K^−1^)
293	−19.8354		
298	−19.4673	−37.46	−60.64
303	−19.1117		
308	−18.7467		
313	−18.5116		

The Royal Society of Chemistry apologises for these errors and any consequent inconvenience to authors and readers.

## Supplementary Material

